# Divorce and Severity of Coronary Artery Disease: A Multicenter Study

**DOI:** 10.1155/2017/4751249

**Published:** 2017-07-24

**Authors:** Amin Daoulah, Mushabab Al-Murayeh, Salem Al-kaabi, Amir Lotfi, Osama E. Elkhateeb, Salem M. Al-Faifi, Saleh Alqahtani, James Stewart, Jon Heavey, William T. Hurley, Mohamed N. Alama, Mazen Faden, Mohamed Al-Shehri, Ali Youssef, Alawi A. Alsheikh-Ali

**Affiliations:** ^1^Section of Adult Cardiology, Cardiovascular Department, King Faisal Specialist Hospital & Research Center, Jeddah, Saudi Arabia; ^2^Cardiovascular Department, Armed Forces Hospital Southern Region, Khamis Mushayt, Saudi Arabia; ^3^Cardiology Department, Zayed Military Hospital, Abu Dhabi, UAE; ^4^Division of Cardiology, Baystate Medical Center, Tufts University School of Medicine, Springfield, MA, USA; ^5^Cardiac Center, King Abdullah Medical City, Holy Capital, Makkah, Saudi Arabia; ^6^Section of Pulmonology, Internal Medicine Department, King Faisal Specialist Hospital & Research Center, Jeddah, Saudi Arabia; ^7^Division of Gastroenterology and Hepatology, The Johns Hopkins Hospital, 1830 East Monument Street, Suite 428, Baltimore, MD 21287, USA; ^8^Anesthesiology Department, King Faisal Specialist Hospital & Research Center, Riyadh, Saudi Arabia; ^9^Emergency Medicine Department, Cleveland Clinic Foundation, Cleveland, OH, USA; ^10^Cardiology Unit, King Abdul Aziz University Hospital, Jeddah, Saudi Arabia; ^11^Anesthesiology Department, King Abdul Aziz University Hospital, Jeddah, Saudi Arabia; ^12^Suez Canal University, Ismailia, Egypt; ^13^College of Medicine, Mohammed Bin Rashid University of Medicine and Health Sciences, Dubai, UAE; ^14^Institute of Cardiac Sciences, Sheikh Khalifa Medical City, Abu Dhabi, UAE

## Abstract

The association between marital status and coronary artery disease (CAD) is supported by numerous epidemiological studies. While divorce may have an adverse effect on cardiac outcomes, the relationship between divorce and severe CAD is unclear. We conducted a multicenter, observational study of consecutive patients undergoing coronary angiography during the period between April 1, 2013, and March 30, 2014. Of 1,068 patients, 124 (12%) were divorced. Divorce was more frequent among women (27%) compared to men (6%). Most divorced patients had been divorced only once (49%), but a subset had been divorced 2 (38%) or ≥3 (12%) times. After adjusting for baseline differences, there was no significant association between divorce and severe CAD in men. In women, there was a significant adjusted association between divorce and severe MVD (OR 2.31 [1.16, 4.59]) or LMD (OR 5.91 [2.19, 15.99]). The modification of the association between divorce and severe CAD by gender was statistically significant for severe LMD (*P*_interaction_ 0.0008) and marginally significant for CAD (*P*_interaction_ 0.05). Among women, there was a significant adjusted association between number of divorces and severe CAD (OR 2.4 [95% CI 1.2, 4.5]), MVD (OR 2.0 [95% CI 1.4, 3.0]), and LMD (OR 3.4 [95% CI 1.9, 5.9]). In conclusion, divorce, particularly multiple divorces, is associated with severe CAD, MVD, and LMD in women but not in men.

## 1. Introduction

Coronary artery disease (CAD) is a major cause of death globally [1, 2]. Modifiable risk factors such as abnormal lipids, smoking, hypertension, diabetes, abdominal obesity, psychosocial factors, lack of daily consumption of fruits and vegetables, and lack of regular physical activity account for the majority of the increased risk for cardiovascular events worldwide in both sexes [3]. Previous cross-sectional studies have examined the association between marital status and health outcomes [4–6]. A number of studies have shown divorce to have a negative impact on cardiovascular health [7–9]. Studies additionally reveal that women suffer more economic and emotional distress as a result of a divorce compared to men [10–13]. A recent study demonstrated that cumulative exposure to divorce increases the risks of myocardial infarction, and women with multiple divorces are at an even higher risk. However, this analysis of myocardial infarction was based on self-reported data [14]. We therefore conducted a study examining the association between divorce and severity of CAD among men and women undergoing coronary angiography for clinical indications.

## 2. Methods

### 2.1. Study Design

The details regarding the design, methods, and endpoints of this multicenter, cross-sectional observational study came from the Polygamy and Risk of Coronary Artery Disease in Men Undergoing Angiography [15]. This study was undertaken to assess the relationship between divorce and severe CAD. It was approved by King Faisal Specialist Hospital & Research Center Institutional Review Board and reviewed for waiver by the institutional review board of each of the participating hospitals. An invitation letter was given to all participants who affirmed verbal consent prior to their enrollment.

### 2.2. Selection Criteria

All patients undergoing coronary angiography for clinical indications were recruited from five hospitals in two Gulf countries (The Kingdom of Saudi Arabia and The United Arab Emirates), during the period between April 1, 2013, and March 30, 2014. These hospitals are tertiary cardiac centers with large patient volumes and advanced cardiac care capabilities. There were no exclusion criteria.

### 2.3. Data Collection

All data were collected prospectively. Two separate data forms, general and angiographic, were filled out by the assigned physician. Both forms were completed prior to the patients discharge from the hospital. All data forms were reviewed by the respective cardiologists and then sent to the principle investigator, who also checked the forms prior to submission for analysis.

*Measures and Variables*. Contents of personal data form (collected through interview) are as follows:

- Demographic data: age, ethnic background
- Physiologic status: hypertension, diabetes, dyslipidemia, and BMI
- Life style: smoking history
- Past medical history: coronary artery disease, percutaneous coronary intervention, coronary artery bypass surgery, cerebral vascular disease, peripheral arterial disease, congestive heart failure, atrial fibrillation, and chronic kidney disease
- Socioeconomic data: occupation (unemployed, private sector, government sector, and self-employed), living in rural or urban area, highest level of education completed (illiterate, secondary school, and higher education), and monthly income (<1300, 1300 to 2600, 2600 to 5300, >5300 USA Dollars)
- Current marital status: divorced (single or multiple times) or not divorced which includes single, married, and widowed status

Contents of angiographic data form (collected from chart review of patient files) are as follows:

- Reason for coronary angiography (elective versus urgent/emergent)
- CAD: number of vessels involved and severity of stenosis
- Treatment (medical versus revascularization)

### 2.4. Definitions

Severe CAD was defined as ≥70% luminal stenosis in a major epicardial vessel or ≥50% stenosis in the left main coronary artery (LMD). Multivessel disease (MVD) was defined as having more than one coronary artery with stenosis ≥70%.

### 2.5. Statistical Analysis

Standard summary statistics were used to describe the cohort. Continuous variables are presented as mean ± standard deviation and were compared across multiple groups using the analysis of variance test. Categorical variables are presented as percentages and compared using the Chi-square test. The associations between divorce and severe CAD, MVD, and LMD were assessed using logistic regression models and quantified with odds ratios. Adjusted regression models included the following explanatory variables: age, gender, community setting (urban versus rural), employment, income level, education level, indication for angiography, and other variables that differed by divorce status in univariate comparisons (*P* < 0.1). All statistical tests were two-sided and significance was set at the conventional *P* value of less than 0.05. No adjustments for multiple comparisons were made.

## 3. Results

### 3.1. Characteristics of Patients and Coronary Angiogram Findings

Overall characteristics of patients and coronary angiogram findings are shown in Table 1. A detailed description can be found in [15].

### 3.2. Patient Characteristics Stratified by Divorce Status

Of the 1,068 patients enrolled, 124 (12%) were divorced. Among the 297 women, 81 were divorced (27%). Among the 771 men, only 43 were divorced (6%), *P* < 0.0001 (Table 1). Most divorced patients had been divorced only once (49%), but some had a history of 2 (38%) or 3 (12%) divorces. One patient had been divorced 4 times. Divorced patients were less likely to have a history of diabetes mellitus or smoking. They were more likely to be unemployed and have a history of atrial fibrillation. Indication for coronary angiogram differed significantly by divorce status with divorced patients more often undergoing coronary angiogram for NSTEACS and less often for STEMI or elective indications. Presence of severe CAD, MVD, or LMD and the subsequent management did not significantly differ by divorce status (Table 1). Additionally, after adjusting for baseline differences and indication for angiogram, a history of divorce was still not significantly associated with severe CAD (OR 0.85 [0.42, 1.73]), MVD (OR 1.76 [1.09, 2.83]), or LMD (OR 1.46 [0.77, 2.76]) (Table 2).

### 3.3. Patient Characteristics Stratified by Divorce Status and Gender

Compared to nondivorced men, divorced men were more likely to be smokers and to have a history of atrial fibrillation and less likely to have LMD on coronary angiogram. Compared to nondivorced women, divorced women were less likely to have diabetes and more likely to have undergone coronary angiogram for NSEACS (Table 1). In univariate analyses, divorced women were more likely to have severe CAD (65% versus. 52%, *P* 0.042), MVD (54% versus. 34%, *P* 0.001), or LMD (17% versus. 3%, *P* < 0.0001) compared to nondivorced women. Consequently, divorced women were more likely to require surgical revascularization (31% versus 10%, *P* < 0.0001) (Table 1). After adjusting for baseline characteristics and indications for coronary angiogram, there was no significant association between divorce and severe CAD in men. In women, there was an association between divorce and severe MVD (OR 2.31 [1.16, 4.59]) or LMD (OR 5.91 [2.19, 15.99]). The modification of the association between divorce and severe CAD by gender was statistically significant for severe LMD (*P*_interaction_ 0.0008) and marginally significant for severe CAD (*P*_interaction_ 0.05) (Table 2). Notably, the modification by gender of the association between divorce and severe CAD or LMD was qualitative such that divorce appeared to have an adverse effect in women and trended toward a decrease in severe CAD in men.

### 3.4. Number of Divorces and Coronary Artery Disease in Women

**Figure 1:**
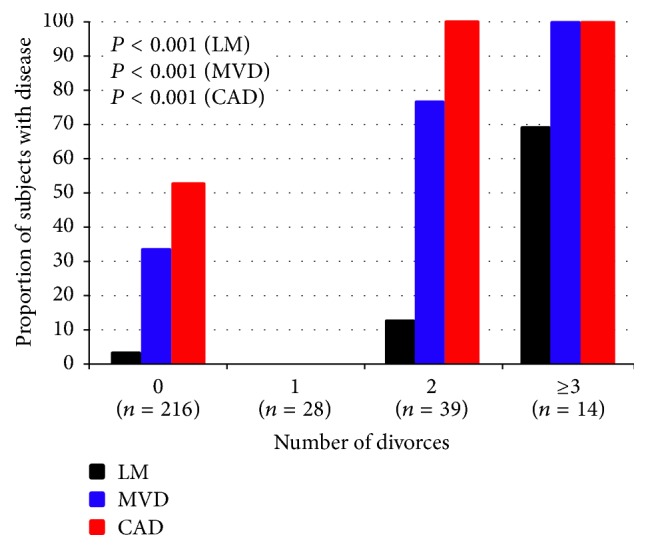
Relationship between number of divorces and severe CAD, MVD, and LMD in women.

**Figure 2:**
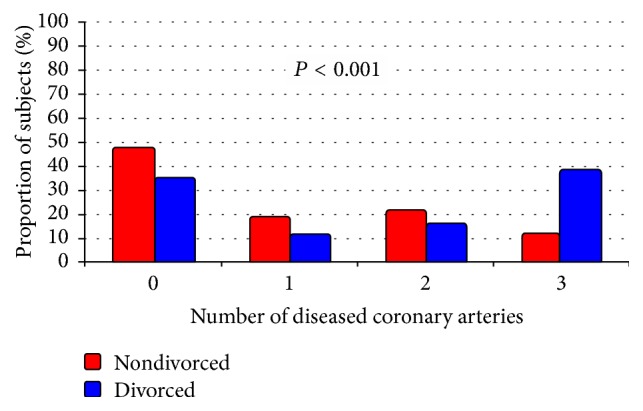
Number of diseased coronary arteries stratified by divorce status in women.

To further assess the relationship between divorce and severe CAD in women, we examined the association between number of divorces and severe CAD in women. In univariate analyses, there was a significant association between the number of divorces and severe CAD, MVD, and LMD in women (Figure 1). The adverse association between divorce and severe CAD appeared to be confined to women with multiple divorces, particularly those with 3 or more divorces, in whom the frequency of severe MVD and LMD was significantly higher than women with 1 or 2 divorces (Figure 1). After adjusting for baseline differences, there remained a significant association between number of divorces and severe CAD (OR 2.4 [95% CI 1.2, 4.5]), MVD (OR 2.0 [95% CI 1.4, 3.0]), and LMD (OR 3.4 [95% CI 1.9, 5.9]). In addition, the number of diseased coronary arteries differed significantly between divorced versus nondivorced women, with the former having a significantly higher rate of severe MVD (38% versus. 12%, *P* < 0.001) (Figure 2).

## 4. Discussion

Our study is the first to look at the association between divorce, including multiple divorces, and severe CAD using coronary angiography in men and women for clinical indications. After adjusting for baseline characteristics and indications for coronary angiogram, a number of observations were made. For women, there was a significant association between divorce, particularly multiple divorces, and severe CAD, MVD, and LMD, while in men, there was no significant association between divorce and severe CAD.

The current statistics from the Ministry of Justice in the Kingdom of Saudi Arabia revealed that approximately 30% of married couples get divorced [16]. Rates of divorce and marriage are difficult to compare globally; many variables lead to differences between these rates, and cohabitation should be considered when comparing global rates. Data from an international report from the Social Trends Institute, the sustainable demographic dividend, demonstrated that the marriage rate in Saudi Arabia is 5.1 per 1000 adult population and in the UK is 4.4 and in the USA is 7.3. The divorce rate is 1.1 per 1000 adult population in Saudi Arabia, 2.4 in the UK, and 3.6 in the USA This may indicate global cultural differences, making it difficult to apply the results of this study globally [17].

Previous cross-sectional studies have examined the association between marital status and health outcomes [4–6]. Molloy et al. studied the extent to which known cardiovascular risk factors contribute to the association between marital status and cardiovascular mortality. They found that health behavior, psychological distress, and metabolic dysregulation contributed to cardiovascular risk in varying degrees [6]. A number of studies have shown divorce to have a negative impact on cardiovascular health [7–9, 14]. Venters et al. found that separated or divorced persons had the highest rates of hospitalization for heart attack and stroke [7]. In another study by Koskenvuo et al., effects of divorce as well as associations with social class were analyzed. They saw higher rates of ischemic heart disease among divorced persons and those in lower social classes [8]. The negative impact of divorce appears to be of limited duration. Adjustment to divorce seems to occur over several years. In women undergoing multiple divorces, the negative impact may have a longer relative duration, having been experienced multiple times over a limited period. Dupre et al. used prospective data to examine the associations between several marital trajectories, mortality, and potential factors contributing to the associations. They found complex associations between marital trajectories and mortality, including significantly higher hazard ratios for men and women currently divorced, women with multiple divorces, and men and women who were recently divorced (within 1–4 years). They found a significantly lower risk of mortality among women divorced for 10 or more years, speculating that the stresses of divorce decline over time due to the ability to adjust to changes in socioeconomic resources, health behaviors, and health status challenges of divorce [9]. Multiple divorces provide the potential for increased financial, emotional, and social stress in needing to maintain multiple households. A recent study demonstrated that cumulative exposure to divorce increased the risks of myocardial infarction and women with multiple divorces were at an even higher risk [14]. However, this analysis of myocardial infarction was based on self-reported data, which may be less accurate than medical evaluation [18–21]. In traditional Middle Eastern societies, divorce produces significant emotional stress for women, more so than men. Such societies are primarily male-dominated with much greater challenges to social status, employment, and housing for divorced women. Community and family support are often minimal or absent for women going through divorce. This is in distinction from western societies, where women have lower levels of emotional stress after divorce than men. Recent societal developments, such as increased education and employment of women, may lessen such stressors, but they remain [22–24]. Diabetes, a traditional risk factor of coronary artery disease, was lower in divorced compared to nondivorced women. Other known traditional risk factors such as smoking, dyslipidemia, and hypertension were not significantly different. On the other hand, unemployment and low income levels, socioeconomic factors associated with coronary artery disease, were higher in divorced women. Multiple interrelated socioeconomic factors, such as unemployment, low income, and divorce status may produce a risk of severe coronary artery disease that meets or exceeds that of traditional risk factors such as diabetes [25].

Several explanations may contribute to the association between divorce, particularly multiple divorces and the severe CAD, MVD, and LMD in women. It is possible that following divorce, women delay seeking care for CAD related symptoms until it has progressed into more severe disease. This may be due to a less robust support system available to divorced women [26–28]. Divorce may additionally have a negative impact on a woman's economic and emotional well-being, which reduces her ability to prevent, detect, and treat cardiovascular-related illness [10–13]. The acute and chronic stress associated with divorce may also play a role [29, 30]. It is likely that biological mechanisms related to the stress of divorce can increase cortisol levels and hemoglobin A1C, may have a role in blood pressure reactivity, reduce sleep time, impair efforts to be physically active, and lead to poor dietary habits [6, 31–36]. Variability in plaque characteristics has recently been shown to correlate with the presentation of CAD. This variability may provide clues to the mechanisms of differential development and presentations of CAD in men and women. For example, culprit plaque rupture and thin-cap fibroatheroma (TCFA) are more prevalent in STEMI patients compared to patients with stable angina, for example. There are multiple factors that affect and increase the risk of plaque rupture. In one meta-analysis, TCFA and smoking were found to be the only predictors for plaque rupture. It would be interesting to compare the plaque burden and plaque rupture between divorced and nondivorced populations. An optical coherence tomography (OCT) study of such a cohort could potentially identify differences in plaque characteristics [37]. In addition, the emotional and economic turmoil a woman faces following a divorce may have negative consequences on adherence to instructions for disease management, including adherence to prescribed medications. This may lead to worsened vascular pathology [38–40]. Although divorce in men appeared to have a trend toward a decrease in severe LMD (*P*_interaction_ 0.0008) and CAD (*P*_interaction_ 0.05), the clinical significance remains unclear. Further studies are required to confirm our findings and to investigate the mechanism underlying these findings to help us identify possible interventions to reduce these risks.

Study strengths are that it is the first to look at the association between divorce, especially multiple divorces, and severe CAD using coronary angiography from Gulf Regions.

*Study Limitations*. Our study had an adequate sample size (1068), but the number of divorced subjects was small (124). The time intervals from divorce to the cardiac catheterization were not recorded; this interval may have influenced the findings. Failing to take into account this time interval and including a significant number of patients with a prior history of CAD (43%) may potentially lead to a reverse causality to the study results. Our study population was selected to undergo coronary angiography if clinically indicated, and, as such, cannot be generalized to all divorced subjects in a healthy population. Unmeasured confounding variables such as dietary habit, physical activity, level of intimacy, inflammatory or stress markers, or other unconsidered variables may have influenced the association.

## 5. Conclusion

Divorce, particularly multiple divorces, is associated with severe CAD, MVD, and LMD in women but not in men. However, future research studies need to measure the time from divorce to clinical presentation and to investigate the mechanism underlying these findings in men and women. Our recommendation from a clinical/public health standpoint is that perhaps programs should be considered to provide greater support to individuals when they become divorced and greater clinical monitoring is indicated.

## Figures and Tables

**Table 1:** Baseline characteristics of the overall cohort stratified by divorce status and gender.

	All patients				Men (*n* = 771)			Women (*n* = 297)		
	All (*n* = 1,068)	No divorce (*n* = 944)	Divorce (*n* = 124)	*P* value	No divorce (*n* = 728)	Divorce (*n* = 43)	*P* value	No divorce (*n* = 216)	Divorce (*n* = 81)	*P* value
Age (yr)	59 ± 13	59 ± 13	58 ± 12	0.1895	59 ± 13	57 ± 15	0.2753	60 ± 13	58 ± 10	0.1178
BMI (kg/m^2^)	28 ± 6	28 ± 6	29 ± 6	0.1415	28 ± 6	28 ± 5	0.6331	29 ± 8	29 ± 7	0.6193
Rural (%)	26	26	23	0.3775	27	35	0.2552	24	16	0.1363
Diabetes mellitus (%)	56	57	48	0.0398	57	44	0.1421	63	49	0.0339
Hypertension (%)	60	59	66	0.0910	57	63	0.4367	67	69	0.6861
Smoking (%)	43	45	34	0.0258	54	70	0.0399	14	15	0.8421
Dyslipidemia (%)	64	65	60	0.2818	66	56	0.1693	60	62	0.7530
Past history (%)										
Coronary artery Disease	43	43	44	0.9011	45	36	0.2439	38	48	0.1116
PCI	23	23	21	0.6652	23	26	0.7092	21	19	0.5977
CABG	6	6	9	0.2455	6	5	0.6836	6	11	0.1356
Atrial fibrillation	6	5	11	0.0075	4	14	0.004	8	10	0.6752
CHF	14	13	17	0.2756	13	14	0.8854	14	19	0.3217
CVA	5	4	7	0.2632	4	7	0.3389	5	6	0.7134
CKD	16	15	19	0.2127	14	23	0.1008	18	17	0.8771
Depression	9	10	7	0.3920	8	0	0.0521	15	11	0.4099
PAD	4	3	6	0.1158	2	5	0.3015	6	6	0.8384
Ethnicity (%)				0.5333			0.2300			0.9455
Arabic Gulf	89	88	91		87	91		92	91	
Arabic non-Gulf	5	6	3		6	0		4	5	
Non-Arabic	6	6	6		7	9		4	4	
Monthly income (%)				0.0902			0.3803			0.6616
<$1300	58	57	69		51	44		78	83	
$1300–2600	24	26	17		29	26		15	12	
$2600–5300	11	11	12		13	26		4	5	
>$5300	7	6	2		7	4		3	0	
Job category (%)				<0.0001			0.1572			0.0722
Jobless	39	35	68		21	28		82	89	
Private sector	13	14	2		18	5		2	1	
Government sector	35	37	21		43	49		15	6	
Self-employed	13	14	9		18	18		1	4	
Education levels (%)				0.2948			0.5954			0.3363
Illiterate	49	48	57		42	49		68	62	
Secondary school	34	34	31		38	32		21	29	
Higher education	17	18	12		20	19		11	9	
Indication for CAG (%)				0.0047			0.3064			0.0017
Elective	48	49	41		46	40		57	42	
NSTEACS	46	45	58		48	58		38	58	
STEMI	6	6	1		6	2		6	0	
Findings on CAG (%)										
Any CAD	68	68	66	0.6272	73	67	0.4232	52	65	0.0426
Single vessel disease	20	22	11		23	12		19	11	
Double vessel disease	25	25	23		26	37		22	16	
Triple vessel disease	23	22	32		24	19		12	38	
Multivessel disease	48	47	55	0.0824	50	56	0.4857	34	54	0.0013
Left main disease	12	11	12	0.7194	13	2	0.0354	3	17	<0.0001
Intervention (%)				0.2298			0.2279			<0.0001
Medical therapy	37	37	34		34	30		50	36	
PCI	45	45	42		46	58		40	33	
CABG	18	18	24		20	12		10	31	

DM, diabetes mellitus; CAD, BMI, body mass index; CAD, coronary artery disease; PCI, percutaneous coronary intervention; CABG, coronary artery bypass grafting; AF, atrial fibrillation; CHF, congestive heart failure; CVA, cerebrovascular accident; CKD, chronic kidney disease; PAD, peripheral arterial disease; $, USA Dollars; Ph.D., a doctor of philosophy; STEMI, ST segment elevation myocardial infarction; NSTEACS, non-ST-segment elevation acute coronary syndromes; CAG, coronary angiography.

**Table 2:** Adjusted association of divorce with severe CAD in the overall cohort and separately in men and women.^*∗*^

	All patients		Men		Women		*P* interaction
	Crude odds ratio	Adjusted odds ratio	Crude odds ratio	Adjusted odds ratio	Crude odds ratio	Adjusted odds ratio	
Any CAD	0.90 [0.60, 1.33]	0.85 [0.42, 1.73]	0.77 [0.40, 1.49]	0.39 [0.14, 1.09]	1.68 [0.98, 2.85]	1.30 [0.51, 3.34]	0.0533
MVD	1.41 [0.97, 2.06]	1.76 [1.09, 2.83]	1.24 [0.67, 2.31]	1.16 [0.57, 2.35]	2.34 [1.39, 3.96]	2.31 [1.16, 4.59]	0.1640
LMD	1.11 [0.63, 1.98]	1.46 [0.77, 2.76]	0.15 [0.02, 1.13]	0.14 [0.02, 1.02]	6.24 [2.42, 16.12]	5.91 [2.19, 15.99]	0.0008

^*∗*^The adjusted regression models included the following explanatory variables: age, gender, community setting (urban versus rural), employment, income level, education level, indication for angiography, and all other variables that differed by divorce status in univariate comparisons with a *P* < 0.1.
